# TMJ Replacement as a System: Results of an International Surgeon Survey on Design, Fixation, Materials, and Digital Workflow

**DOI:** 10.3390/cmtr19020024

**Published:** 2026-05-19

**Authors:** Sergio Olate, Wenko Smolka, Ricky Kumar, Rüdiger Zimmerer, Zachary S. Peacock

**Affiliations:** 1 Center for Research in Morphology and Surgery (CEMyQ), University of La Frontera, Temuco 4811230, Chile; 2 Department of Oral Diagnosis, Division of Oral and Maxillofacial Surgery, State University of Campinas, Piracicaba, São Paulo 13414-903, Brazil; 3 Department of Oral and Maxillofacial Surgery and Facial Plastic Surgery, University Hospital LMU Munich, 81377 Munich, Germany; wenko.smolka@med.uni-muenchen.de; 4 Royal Children’s Hospital Melbourne, Parkville 3052, Australia; rickumar@gmail.com; 5 Department of Oral and Maxillofacial Surgery, University Hospital Essen, 45239 Essen, Germany; ruediger.zimmerer@uk-essen.de; 6 Department of Oral and Maxillofacial Surgery, Harvard School of Dental Medicine; Massachusetts General Hospital, Boston, MA 02115, USA; zpeacock@mgb.org

**Keywords:** temporomandibular joint, arthroplasty, temporomandibular joint disease, survey

## Abstract

The evolution of TMJ-replacements has been driven by advancements in personalization, materials and digital planning technologies. However, there is little data on how surgeons perceive the prosthetic systems and their limitations in practice. The perspective from surgeons is critical to identifying the gap between current technology and clinical need. Using an international, structured survey based on a modified Delphi methodology, including expert validation, pilot testing and global distribution through professional networks, the status of the TMJ-replacement ecosystem was obtained. The questions covered aspects such as the workflow, the integration of components, the design features, the use of materials, the fixation strategy for each component, and usability of surgical guides. The responses were quantitatively analyzed using descriptive statistics. 250 surgeons were included; the majority of surgeons preferred to have all elements of the TMJ-replacement system provided by a single system or company (i.e., the prosthesis and plates), with 86.0% of surgeons indicating this preference and 71.2% of surgeons indicating that having access to STL files for use in surgical navigation is an important feature. Approximately 88.4% of surgeons indicated that flexibility in the design of the prosthesis is important, while 61.6% of respondents indicated that design issues contribute to failures of TMJ prostheses. Issues related to the fixation of the prosthetic components, particularly screw loosening, were also commonly reported by surgeons with respect to failure. Surgeons also indicated concerns with regard to the bulkiness (73.2%) and the fit accuracy (68.4%) of the surgical guides. In terms of material selection, 78.0% of respondents preferred to be able to choose the type of metal alloy for the TMJ replacement system. TMJ replacement is viewed by surgeons as a system-dependent procedure that requires the integration of design, materials, fixation and digital workflow. The currently available prosthetic solutions only partially meet the needs of surgeons.

## 1. Introduction

Total temporomandibular joint (TMJ) replacement has become an established reconstructive option for patients with advanced joint degeneration, ankylosis, tumor-related defects, and failed prior reconstructions. Advances in imaging, biomaterials, computer-assisted planning, and manufacturing technologies have improved the precision and predictability of these procedures [1,2,3]. Healthcare systems need to find reliable solutions for complex functional conditions that significantly affect quality of life [3,4].

TMJ reconstruction with alloplastic prostheses depends on four interconnected elements: the patient, the surgeon, the industry, and the healthcare system. The patient presents highly variable anatomical and pathological conditions. The surgeon is responsible for translating planning into execution under demanding clinical circumstances. The industry provides the implants, planning tools, and support systems. The public or private healthcare system influences access to technology, regulatory pathways, and logistics. The interaction among these elements ultimately determines the effectiveness and safety of TMJ replacement, and an efficient surgical workflow [5,6].

Despite significant progress in prosthetic design and manufacturing, important limitations persist. Prosthetic systems must adapt to complex anatomy, allow stable fixation in compromised bone, and remain compatible with surgical workflows that vary across institutions and countries [4,7].

For many surgeons, TMJ replacement remains a low-frequency but high-complexity procedure and depends on manufacturers for accurate planning platforms, predictable timelines, and intraoperative support [8]. Even small inefficiencies related to implant design, fixation, digital planning, or surgical guides can increase the risk of complications [9].

Although current systems are functional, surgeons continue to encounter challenges related to the use of TMJ prosthesis; there is a gap between the capabilities of available prosthetic systems and the needs for clinical practice. An evaluation of the surgeon perspectives is therefore essential to identify these gaps and to understand how technological advances translate into clinical usability [10]. Such information is critical for improving the ecosystem that surrounds TMJ replacement.

Understanding these perspectives is essential to guide improvements in prosthetic design, enhance integration of digital workflows, and support more efficient surgical decision-making. Ultimately, this approach may contribute to improved clinical outcomes and the evolution of TMJ replacement as a system-based procedure.

The objective of this study was to assess the perspectives of maxillofacial surgeons worldwide regarding current TMJ prosthetic systems, focusing on clinical workflow, design and material considerations, fixation strategies, and usability.

## 2. Materials and Methods

This was an internationally based, cross-sectionally study to establish what surgeons believe about TMJ prosthetic systems. A modified Delphi approach was used to construct the methodology. Given the exploratory and descriptive nature of this study, no formal sample size calculation was performed. The aim was to obtain a broad representation of surgeon perspectives across different regions and practice settings.

Five experienced maxillofacial surgeons with extensive backgrounds in TMJ reconstructive surgery were consulted to define the areas of focus that would be studied as part of this investigation. The thematic domains identified during this process included surgical experience and clinical context, surgical workflow, prosthesis design, material aspects, fixation methods, failure mechanisms, and usability of surgical guides. From the thematic domains, a first draft of the questionnaires and answers was developed.

After developing the initial drafts, a review was held to assess whether the draft questionnaire could be universally understood by all surgeons across the globe regardless of location or culture. Eleven surgeons from different geographic regions (e.g., Europe, Latin America, USA, and Asia-Pacific) participating in this review assessed the clarity and interpretability of the questions across different clinical settings. Any feedback received during this review process was utilized to improve the survey.

The third phase of the expert review process involved reviewing the survey to eliminate redundant questions and to further optimize the logical flow of the survey (10 surgeons involved in this process). After completing this phase, pilot testing was initiated involving twelve surgeons who reviewed the survey to assess its usability, the time required to complete it, and the comprehensibility of the questions. Minor modifications were made to the survey after receiving feedback from the pilot testing program.

Formal psychometric validation and reliability testing were not performed, as the primary aim of this study was exploratory and descriptive. However, the questionnaire underwent expert review, iterative refinement, semantic evaluation across regions, and pilot testing to enhance clarity, consistency, and content validity.

**Figure 1:**
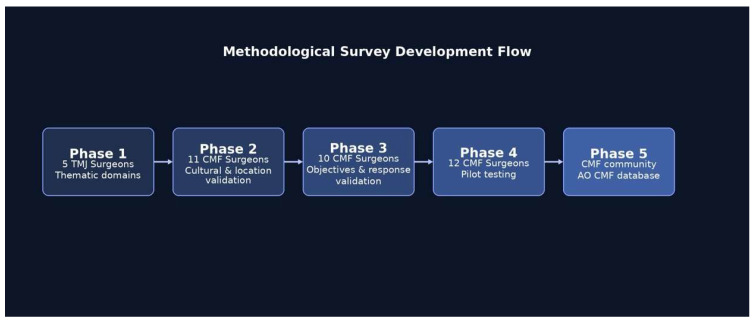
Methodological framework for survey development in temporomandibular joint surgery. It should be noted that the AO CMF database comprises approximately 4000 surgeons. However, the survey was specifically directed toward surgeons involved in temporomandibular joint (TMJ) reconstruction, and participants were asked to confirm that they met this criterion prior to initiating the survey.

The final version of the survey contained twenty-five questions using categorical response options to enable objective quantification. Question types included multiple-choice and Likert scale format dependent upon the type of data being collected. A schematic representation of the survey development process, including expert review, pilot testing, and survey distribution, is presented in Figure 1.

The survey was distributed worldwide over a period of one month using direct e-mail solicitations and professional networking opportunities, including disseminating through channels of the AO Foundation. Only surgeons with experience in TMJ replacement were included in this study. Participation was entirely voluntary and anonymous; no incentive was offered to those participating.

All responses to the survey were collected electronically and placed in a database. Only surveys containing fully completed responses were entered into the analysis. Descriptive statistical analyses were performed on the data to obtain absolute frequency distributions and percent values. The results were categorized by thematic domain and included: clinical context, workflow, design, materials, fixation, and usability.

The data collection complied with ethical guidelines for conducting research. This study was conducted in accordance with the Declaration of Helsinki and the U.S. Common Rule (45 CFR 46.104[d][2]). No personal or identifiable information was collected, and all data were handled anonymously and confidentially throughout the study.

The full questionnaire is provided as a Supplementary Material (Supplementary File S1) to ensure transparency and reproducibility.

## 3. Results

250 completed responses from maxillofacial surgeons around the world were included in the analysis. Due to the distribution of the survey through professional networks and open dissemination channels using the AO database, it was not possible to determine the total number of recipients or calculate a response rate. This represents a limitation of the study.

### 3.1. Surgeons Profile and Clinical Context

Surgeons represented a broad international distribution, with the largest proportions of responses obtained from the Asia-Pacific (28.4%), Europe and Southern Africa (26.4%), and Latin America (20.8%), followed by North America (12.4%) and the Middle East and Northern Africa (12.0%). The most frequent countries were India (12.8%), the United States (10.0%), and Brazil (7.2%). Regarding the practice setting, 49.6% were working primarily in university or academic hospitals, 25.2% in private practice, and 21.6% in public hospitals. Most participants identified their professional status as specialist or consultant (83.2%); 34.8% showed more than 20 years in practice, while 25.6% presented 11–20 years, 18.8% reported 5–10 years. Most surgeons (64.8%) reported performing between 1 and 4 TMJ replacements per year; 18.4% reported performing 5–10 cases annually, 7.6% reported 11–20 cases and 8.4% performed more than 20 TMJ replacements per year (Table 1).

### 3.2. Surgical Workflow, System Integration, and Digital Support

A majority of surgeons (86.0%) considered it somewhat or extremely important to have a single company responsible for both the design and manufacture of the TMJ prosthesis and the orthognathic or maxillofacial fixation plates used during the same procedure. Regarding acceptable production timelines after data upload, 84.0% of surgeons considered a lead time of 6 weeks or less to be acceptable, with 37.2% selecting 2 weeks and 46.8% selecting 6 weeks as the maximum acceptable timeframe.

**Figure 2:**
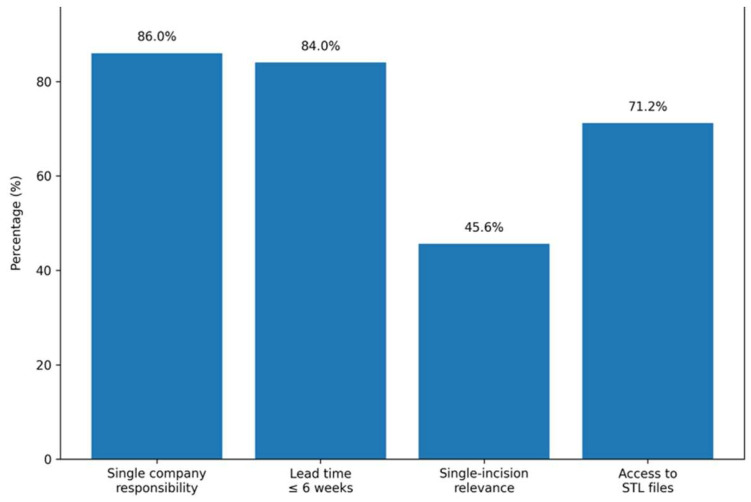
Main responses obtained in domain surgical workflow, system integration, and digital support. Data are presented as percentages (%) of the total sample (n = 250).

The digital resources for intraoperative navigation were also emphasized, with 71.2% of surgeons rating access to the STL files as somewhat or extremely important. In relation to surgical treatment, 45.6% of surgeons considered the possibility for a single-incision approach for some cases to be somewhat or extremely important, while 31.2% reported a neutral position (Figure 2).

### 3.3. Prosthesis Design and Biomechanical Considerations

Flexibility in design was considered somewhat or extremely important by 88.4% of surgeons, and only 1.2% considered it not important. Regarding condylar head morphology, 42.0% of surgeons chose an anatomic shape, followed by spheroid (28.8%) and ellipsoid (26.4%) designs. In fossa design, critical aspects were posterior stop (48.4%), followed by anterior stops and anatomic shape, each selected by 36.8%.

**Figure 3:**
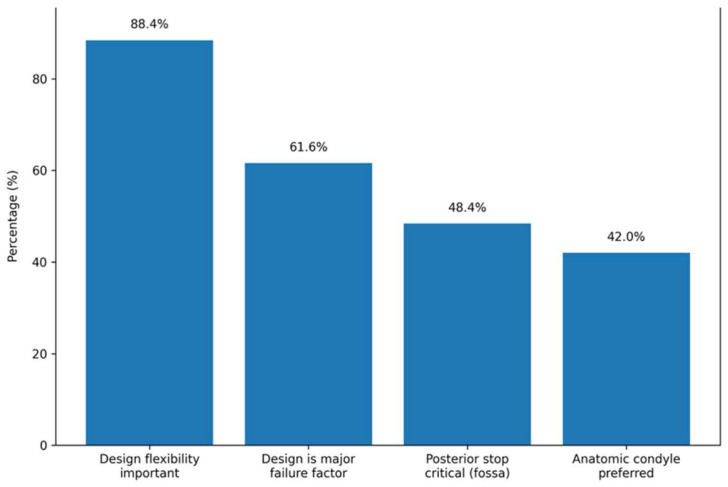
Main responses obtained in domain prosthesis design and biomechanical considerations. Data are presented as percentages (%) of the total sample (n = 250).

The perceived impact of prosthesis design on system failure was important; 61.6% of surgeons considered design to be one of the most important factors contributing to failure, while only 3.2% considered design unrelated to failure (Figure 3).

### 3.4. Materials, Fixation Strategies, and Failure Mechanisms

The ability to choose the alloy for the condylar component was considered somewhat or extremely important by 78.0% of surgeons, while 19.2% reported a neutral position; 66.8% of surgeons rated the use of a titanium-based fossa as somewhat or extremely important.

Regarding perceived causes of component failure, fracture or loosening of screws (24.4%) and design error (24.0%) were the most common variables, followed by surgical error (19.2%). In cases of ramus-related failure, a lack of adaptability at the level of condylar neck osteotomy was selected by 31.6% of surgeons. Failure in the adaptation of the lower part of the implant (28.4%) and screw torque or stability issues (25.6%) were also reported.

For fossa-related failure, screw loosening or fracture was the most frequent cause (32.0%), followed by polyethylene wear (24.8%) and surgical error (18.4%).

**Figure 4:**
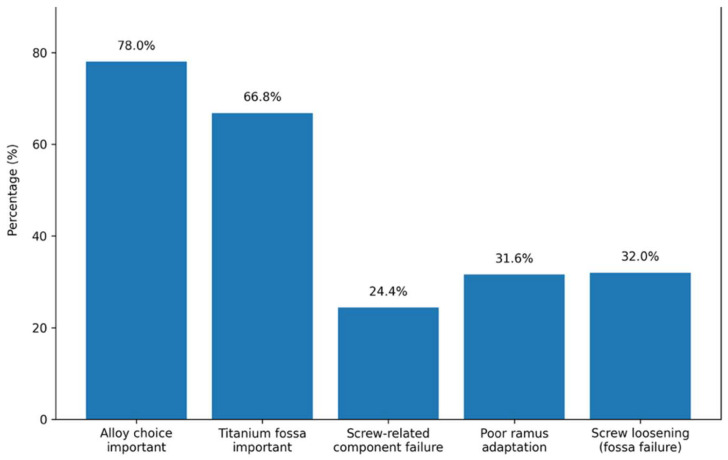
Main responses obtained in domain materials, fixation strategies, and failure mechanisms. Data are presented as percentages (%) of the total sample (n = 250).

In terms of fixation strategies, 40.0% of surgeons considered four screws to be the minimum required for fossa fixation, while 13.2% chose five screws and 12.4% chose three screws. For ramus fixation, six screws were the most frequently selected (24.8%), followed by five screws (14.4%) and four screws (12.4%) (Figure 4).

### 3.5. Surgical Guides and Usability

Concerns related to surgical guides were reported by surgeons. Moderate to major concern regarding the material used to fabricate surgical guides was chosen by 53.6% of surgeons.

**Figure 5:**
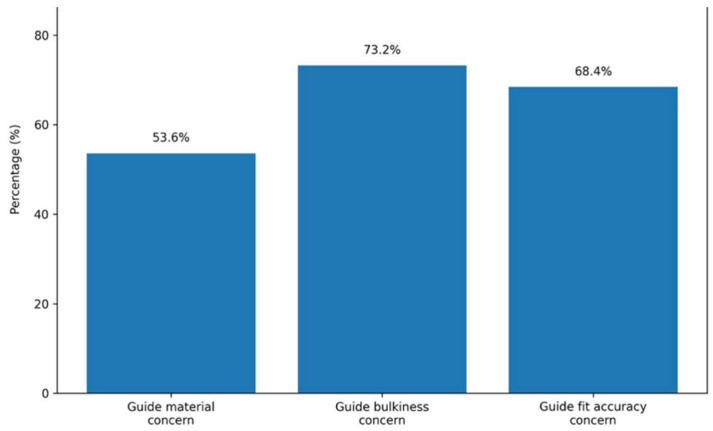
Main responses obtained in domain surgical guides and usability constraints. Data are presented as percentages (%) of the total sample (n = 250).

In terms of guide bulkiness, 73.2% of surgeons reported moderate to major concern. Issues related to fit accuracy were reported by 68.4% of surgeons at a moderate to major level (Figure 5).

### 3.6. TMJ Prosthesis Failure

61.6% of surgeons considered prosthesis design to be one of the most important factors contributing to system failure.

**Figure 6:**
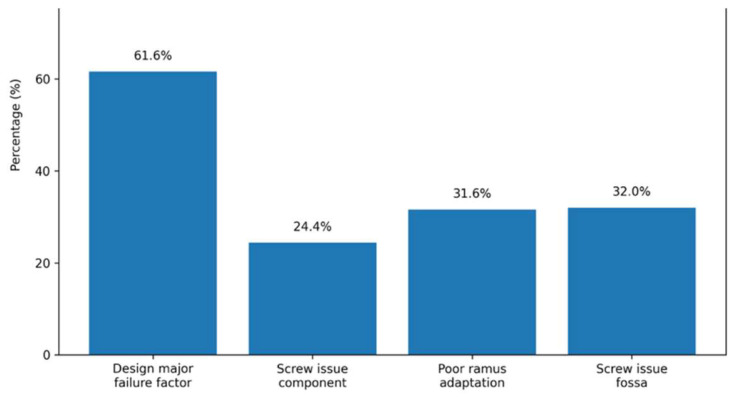
Main responses obtained in domain overview of key reported contributors to TMJ prosthesis failure. Data are presented as percentages (%) of the total sample (n = 250).

At the component level, fracture or loosening of screws was the most frequently reported cause of failure (24.4%). In ramus-related failure, the lack of adaptability of the prosthesis at the condylar neck osteotomy level was selected by 31.6% of surgeons. For fossa-related failure, screw loosening or fracture was identified as the most common cause (32.0%) (Figure 6).

## 4. Discussion

The study aimed to evaluate the perceptions of surgeons concerning the current status of TMJ prosthetic systems and the existing gaps between what is available and what is required from surgeons who perform TMJ-replacements. As a survey-based study, the findings are subject to inherent response and perception bias, as they reflect subjective opinions rather than objective clinical measurements. Surgeons perceive TMJ replacement as a system-dependent procedure and need all components of a TMJ replacement system (design, material, fixation and digital workflow) to work together.

The methodologic strength of this study is the systematic development of the survey tool and the scope of the survey’s international application. The questionnaire was developed using a modified Delphi technique of successive rounds of expert reviews, semantic validation of terms in each region and pilot testing before dissemination to ensure clarity, relevance and internal consistency of the items [11]. Using the modified Delphi technique to develop the survey minimized the ambiguity associated with interpreting the results by making sure that all experts used the same terminology and conceptual framework when responding to the survey. Additionally, using categories for surgeons made it possible to objectively quantify the responses [12].

The sample of 250 surgeons included a geographically diverse population of maxillofacial surgeons who practiced in academia, public and private sectors. Most of the participants were either specialists or consultants with clinical experience, and although several surgeons reported performing TMJ replacement at low to moderate frequency per year, it is essential to note that this is reflective of the actual clinical usage of TMJ prosthetic surgery worldwide [13]. Together the combination of experienced surgeons, international representation and realistic procedural exposure provide a representative view of how TMJ prosthetic systems are being used in clinical practice [14,15].

System integration was found to be a significant concern, with 86.0% of surgeons reporting that they value having one company responsible for both the TMJ prosthesis and fixation systems; and 71.2% of surgeons reporting that access to STL files for intra-operative navigation is very important. 84.0% of respondents reported that a production lead time of six weeks or less is acceptable. These results illustrate that TMJ replacement is viewed not solely as an isolated implant procedure, but as a systemic process including planning, manufacturing and surgical execution. There are numerous reports documenting that over 65% of TMJ-replacement cases are performed in conjunction with orthognathic surgery, wherein the use of other materials beyond the joint prosthesis plays a critical role in achieving comprehensive case resolution [3,16]. Therefore, surgeons wish to utilize a single platform or company to achieve comprehensive case resolution through the integration of all system components. However, differences in access to TMJ prosthetic systems across healthcare settings, including academic, public, and private institutions, as well as cost-related factors, were not specifically evaluated and may influence surgeon responses.

These views are consistent with the rationale outlined by Mercuri [17,18] and the long-term clinical experiences of Wolford [1] which emphasize the necessity for close collaboration between surgeon and manufacturer throughout the planning to production continuum. The emphasis placed on digital resources and workflow simplification in the survey also show changes occurring in craniomaxillofacial surgery, where virtual planning and digital integration have become core components of surgical preparation [19]. Contemporary surgical innovation increasingly depends on system level integration rather than on isolated device features [20], which is a concept clearly presented in the results of this survey.

Design and biomechanical performance of the prosthesis were also identified as critical factors; 88% of surgeons rated design flexibility as important, and 61% of surgeons indicated that prosthesis design is an important contributor to system failure. The difference between clinical indications and anatomy necessitates implants that are tailored during design and manufacture. For example, longer prostheses require different structural support than smaller prostheses [21], which translates into unique surgical requirements for each patient.

The individualization of surgery is also observed in the surgical approach; 45.6% of surgeons were positive regarding the reduction in surgical access to a single incision. Although this percentage can be viewed as moderate, there are studies [22] demonstrating that the utilization of a single surgical access improves postoperative recovery, which suggests a potential short-term trend towards minimally invasive TMJ-replacement surgery. Similarly, another article [23] describes intra-oral approaches as alternatives to transcervical or submandibular accesses, utilizing the same minimally invasive surgical principle.

Preferences related to the characteristics of the condyle, the fossa elements and related fixation indicated that surgeons view the prosthesis as a biomechanical unit that can be tailored to meet both anatomical and functional demands. Support for this perception comes from finite element analysis that present high levels of stress concentration at the fixation areas of the condylar unit, and thus highlights the significance of geometry and fixation on the mechanical integrity of the implant [24,25]. The variability in preferences regarding condylar and fossa design further supports the concept that TMJ prosthesis selection is highly surgeon-dependent and must be adapted to individual anatomical and clinical conditions.

Biomechanical modeling has demonstrated that masticatory loading and peak bite forces produce stress patterns across the various components of the implant and surrounding bone [26,27]. Research using PSI have shown lower levels of stress distribution and improved load transmission through the use of customized devices compared to standard designs [28].

The perception that prosthesis design is a major contributor to failure may be explained by the complex biomechanical environment of the TMJ. Implant geometry, load distribution, and fixation strategy are closely interrelated, and inadequate adaptation of the prosthesis may lead to stress concentration, screw loosening, or mechanical fatigue. Previous biomechanical and finite element studies have demonstrated that variations in implant design significantly influence stress-distribution patterns and overall mechanical stability [24,25,26,27,28,29]. These findings support the relevance of design considerations in clinical outcomes; although biomechanical stress patterns were not directly assessed, existing literature supports their role in implant performance.

The majority of surgeons (78%) stated that the ability to select the alloy for the condylar component was important and 66.8% believed that a titanium based fossa was very important. The views expressed by the surgeons were supported by studies that have shown that the micro-structure and electrochemical properties of the different alloys used in TMJ prostheses vary significantly and can affect long-term performance and local tissue response to the implant [30]. Yuceer-Cetiner et al. [30], demonstrated that different alloys show significant differences in load bearing support, and suggested that alloy selection is not simply an engineering decision, and can be related to clinical confidence and reliability. Alloy immunogenicity is also an additional factor that should be considered when evaluating potential prosthesis failure due to metal hypersensitivity.

The complications seen in TMJ prostheses are well documented [9]. Surgeons’ perceptions indicate that fixation-related problems were cited as contributing to failure at all levels of the implant, and surgeons provided clear indications of what they believe to be the optimal number of screws required for stable fixation. These findings are consistent with other analyses [25,31], which have identified fixation problems as one of the leading causes of TMJ prosthesis failure. These data suggest that the number of screws, the length, placement, orientation and method of screw insertion should be evaluated to assess the usability and reliability of the fixation.

Another area of concern was the usability of surgical guides in current systems. Surgeons reported significant concerns regarding guide bulkiness (73.2%) and accuracy of fit (68.4%), suggesting persistent challenges in translating digital planning into precise intraoperative execution. This finding is particularly relevant when compared with existing commercial guide systems, which, despite advances in CAD/CAM, are still perceived as bulky, especially in anatomically constrained regions such as the mandibular ramus and TMJ area. Several commercially available systems prioritize structural rigidity and fixation stability, often at the expense of reduced profile and ease of intraoperative handling, which may contribute to soft tissue tension, limited surgical access, and increased operative complexity.

This observation is consistent with previous reports [3,32], which have highlighted the need for fully integrated digital workflows to improve coordination in TMJ surgery, particularly when combined with orthognathic procedures. Surgical guides that enable precise osteotomies, accurate positioning, and stable fixation are essential in these complex interventions. Therefore, surgical guide design should integrate ergonomic principles, minimizing bulk without compromising structural integrity, while ensuring precise adaptation to the anatomical constraints and procedural demands of each case. These limitations highlight the need for further refinement in guide design to enhance usability and surgical precision.

Design, material, fixation and surgical procedure in PSI are viewed with precision, material performance, and intraoperative accuracy; however, they are variables that together determine the success of TMJ prosthetics. Kanatsios et al., [33] demonstrated that patients receiving custom-made TMJ prostheses exhibit better clinical outcomes compared to patients receiving stock prostheses, and Vignesh et al., [34] demonstrated that patients treated with PSI and precisely executed surgical techniques exhibit more favorable stress distributions. For this reason, it is important to highlight that surgeons view these elements as weaknesses in some of the current systems on the market.

Additionally, the results of this study demonstrate the necessity for ongoing education and training for surgeons who perform TMJ replacement. With the increasing dependence of TMJ implants on digital planning, material selection, biomechanical understanding, and the integration of a series of technological tools and workflows, the role of the surgeon extends beyond the technical execution of the surgical procedure to the application of design principles, fixation strategies, and technological resources. Although some studies have shown that exposure in training programs for TMJ replacement is sufficient [35], it will be necessary to integrate the entire workflow and the continued technological advancements of these processes.

This study has several limitations; first, although the sample size comprises 250 surgeons from a variety of geographic locations and practice settings, it is likely that the responses may have been influenced by the individual clinical experience, the local healthcare system, and the degree of exposure to various TMJ prosthetic platforms; the variability in surgeon experience, clinical indications, and technical preferences represents a real-world characteristic of TMJ-replacement practice and may contribute to the heterogeneity of responses. However, this diversity also strengthens the relevance of the findings by reflecting current global clinical practices.

Second, the fact that many of the surgeons in the sample have relatively low to moderate annual volumes of TMJ replacements may also impact the perceptions regarding workflow, fixation and guide usability. Since the responses to the survey questions were categorized into some options, the surgeons may not have had the opportunity to express their opinions for a more accurate representation of their perceptions.

Additionally, a potential self-selection bias should be considered, as surgeons with stronger opinions, greater experience, or higher interest in TMJ replacement may have been more likely to participate, potentially influencing the distribution of responses.

Another limitation of this study is the inability to accurately determine the true denominator. Although the survey was distributed through an organized database comprising more than 4000 craniomaxillofacial surgeons, it is likely that a proportion of recipients are not involved in temporomandibular joint (TMJ) surgery and, therefore, were not eligible to respond to this survey. Additionally, it cannot be excluded that some email addresses may be outdated or incorrect, or that the survey invitation may have been filtered as spam, preventing potential participants from accessing the questionnaire. For these reasons, it was not feasible to precisely define the denominator population in this study.

Lastly, as with any study based on perceptions, the results do not represent objective measures of clinical performance or outcome, and therefore must be interpreted cautiously.

## 5. Conclusions

TMJ replacement is increasingly perceived by surgeons as a system-dependent procedure requiring integration between prosthesis design, material selection, fixation strategies, and digital workflow. In this international survey, 86.0% of surgeons preferred integrated systems provided by a single manufacturer, 88.4% valued flexibility in prosthesis design, and 78.0% considered the ability to select the alloy composition as important. At the same time, relevant limitations were identified, with 73.2% of surgeons reporting concerns regarding the bulkiness of surgical guides and 68.4% regarding fit accuracy. Notably, 61.6% of respondents identified prosthesis design as a major contributor to system failure, emphasizing the critical role of biomechanical performance and implant adaptation in clinical outcomes.

These findings highlight clinically relevant gaps that may increase the risk of mechanical complications, including screw loosening, suboptimal load distribution, and intraoperative inaccuracies. To address these limitations, manufacturers should prioritize the development of integrated platforms combining prosthesis design, fixation systems, and digital planning tools, with particular attention to improving guide usability and precision. Surgeons should continue advancing their expertise in digital workflows and system-based approaches, while future research should aim to correlate these perceptions with objective clinical outcomes. Ultimately, the evolution of TMJ prosthetic systems will depend on the convergence of engineering innovation, digital technologies, and clinical expertise, supporting a more predictable, efficient, and patient-specific reconstructive paradigm.

## Figures and Tables

**Table 1:** Distribution and characteristics of the group of 250 surgeons who responded to the TMJ-replacement survey.

Variable	Category	N (%)
Region of practice	Asia-Pacific	71 (28.4%)
	Europe and Southern Africa	66 (26.4%)
	Latin America	52 (20.8%)
	North America	31 (12.4%)
	Middle East and Northern Africa	30 (12.0%)
Practice setting	University/Academic hospital	124 (49.6%)
	Private practice	63 (25.2%)
	Public hospital	54 (21.6%)
	Other	9 (3.6%)
Professional status	Specialist/Consultant	208 (83.2%)
	Resident	19 (7.6%)
	Fellow	14 (5.6%)
	Other	9 (3.6%)
Years in clinical practice	More than 20 years	87 (34.8%)
	11–20 years	64 (25.6%)
	5–10 years	47 (18.8%)
	Less than 5 years	52 (20.8%)
Annual TMJ replacements	1–4 cases/year	162 (64.8%)
	5–10 cases/year	46 (18.4%)
	11–20 cases/year	19 (7.6%)
	More than 20 cases/year	21 (8.4%)

Note: N Number of surgeons who answered the questions.

## Data Availability

The data presented in this study are available on request from the corresponding author.

## Supplementary Materials

The following supporting information can be downloaded at: https://www.mdpi.com/article/10.3390/cmtr19020024/s1, File S1.
