# Supplementary material for: TMJ Replacement as a System: Results of an International Surgeon Survey on Design, Fixation, Materials, and Digital Workflow
Source: Craniomaxillofac Trauma Reconstr. 2026 May 19;19(2):24. doi: 10.3390/cmtr19020024 (PMC13214619; doi:10.3390/cmtr19020024)
Supplement: Supplementary file 1 [file cmtr-19-00024-s001.zip › cmtr-4196070-supplementary.pdf]

Supplemental File S1

Systematic classification of survey questions into thematic areas. Each thematic area is defined and the corresponding questions assigned to it (e.g., respondent profile, market structure, design considerations, materials, fixation and failure mechanisms, surgical guides, digital workflow)

1. Sample Characterization / Respondent Profile

Objective: To define the demographic, geographic, and professional profile of the respondents, ensuring that the survey results reflect a relevant, experienced, and globally representative population.

Questions included:

- Q1. Region of practice
  Objective: To assess the global distribution of respondents and confirm international representation.
- Q2. Country of practice
  Objective: To identify key national markets and contextualize regional influence on responses.
- Q3. Primary practice setting
  Objective: To understand the clinical environment influencing adoption patterns.
- Q4. Annual TMJ replacement volume
  Objective: To distinguish between low-, medium-, and high-volume surgeons.
- Q5. Years in clinical practice
  Objective: To evaluate professional maturity and long-term exposure.
- Q6. Career status
  Objective: To confirm that responses primarily reflect senior decision-makers.

2. Market Structure, Company Model, and Digital Workflow Integration

Objective: To evaluate surgeon expectations regarding industry structure, accountability, speed, and integration of digital tools into the surgical workflow.

Questions included:

- Q7. Single company responsibility for prosthesis and plates
  Objective: To assess demand for vertically integrated supplier models.
- Q8. Acceptable production lead time
  Objective: To define market expectations regarding manufacturing speed.

- Q18. Importance of a single-incision approach for simpler cases
  Objective: To evaluate workflow flexibility and openness to procedural simplification within integrated systems.
- Q25. Access to company STL files for navigation
  Objective: To assess expectations regarding digital openness and intraoperative navigation support.

3. Prosthesis Design Philosophy, Adaptability, and Satisfaction with Current Solutions

Objective: To understand surgeon priorities related to prosthetic geometry, flexibility, performance, and overall satisfaction with existing designs.

Questions included:

- Q9. Importance of design flexibility
  Objective: To assess the value placed on adaptable prosthetic features.
- Q10. Preferred condylar head shape
  Objective: To explore design preferences and kinematic expectations.
- Q11. Critical fossa design elements
  Objective: To identify essential functional and anatomical features.
- Q14. Impact of design on system failure
  Objective: To quantify the perceived relationship between design and failure risk.
- Q22. Satisfaction with fossa size and volume
  Objective: To evaluate how well current designs meet anatomical and functional expectations.

4. Materials and Component Construction

Objective: To evaluate the perceived relevance of material selection and its impact on performance and reliability.

Questions included:

- Q12. Importance of choosing condylar alloy
  Objective: To assess demand for material customization.
- Q17. Importance of a titanium-based fossa
  Objective: To understand surgeon perception of material standards.

5. Fixation Strategy and Mechanical Reliability

Objective: To identify surgeon expectations regarding fixation robustness, failure mechanisms, and mechanical stability.

Questions included:

- Q13. Main cause of component failure
  Objective: To identify dominant perceived failure drivers.
- Q15. Causes of failure in the ramus area
  Objective: To analyze ramus-specific mechanical and adaptation challenges.
- Q16. Causes of failure in the fossa
  Objective: To evaluate fossa-side fixation and wear issues.
- Q23. Minimum number of screws for fossa fixation
  Objective: To identify reference standards and variability in fossa fixation.
- Q24. Minimum number of screws for ramus fixation
  Objective: To assess fixation strategies in a mechanically demanding region.

6. Surgical Guides, Precision, and Usability

Objective: To evaluate limitations of current surgical guides and identify opportunities to improve precision and ergonomics.

Questions included:

- Q19. Concerns regarding guide material
  Objective: To assess confidence in material performance.
- Q20. Concerns regarding guide bulkiness
  Objective: To identify ergonomic and access-related limitations.
- Q21. Concerns regarding fit accuracy
  Objective: To evaluate perceived reliability of guided workflows.
